# Comparison of quantitative and semi-quantitative sampling methodologies for biomonitoring of Mediterranean streams using benthic macroinvertebrates: a case study from Greece

**DOI:** 10.1007/s10661-022-10620-9

**Published:** 2022-11-03

**Authors:** Eleana Kazila, Chrysoula Ntislidou, Catherina Voreadou

**Affiliations:** 1 Natural History Museum of Crete, University of Crete, 71409 Heraklion Crete, Greece; 2 Department of Zoology, School of Biology, Aristotle University of Thessaloniki, 54124 Thessaloniki, Greece

**Keywords:** Freshwater community metrics, Hellenic Evaluation system 2, Sampling efficiency, Shovel sampler, D-frame net, Water Framework Directive

## Abstract

In Greece, the implementation of the Water Framework Directive for rivers is based mainly on benthic macroinvertebrates and uses a semi-quantitative method with a D-frame net, which is certified by the International Organization for Standardization. Before the official adoption of this method, a quantitative shovel sampler (“Cretan shovel”) was used in southern Greece (Crete), which has been implemented for almost three decades due to the specific river habitats found in Crete (e.g., seasonal flow, narrow riverbeds, and coarse substrates). In this study, we compared community metrics, diversity indices, feeding groups, locomotion types, and ecological quality derived from data collected using timed semi-quantitative kick samples and quantitative shovel samples collected from the same sites simultaneously. In total, 20 samples from the north and south of Greece were collected. The majority of community metrics, diversity indices, and traits were comparable between samplers. However, there were statistically significant differences in the relative abundance of Ephemeroptera, Plecoptera, and Trichoptera, passive filterers’ and the (semi) sessil groups, and Pielou’s index. Most differences in the ecological quality between the kick and shovel samples were observed in 50% of the sites in northern Greece because the shovel is less effective at capturing motile zoobenthos. The ecological quality assessment in Crete by the Cretan shovel is comparable with the D-frame net in 75% of the samples. Thus, the Cretan shovel could efficiently sample the Cretan streams, which are characterized by coarse, narrow, turbulent, and hydrologically fluctuating river habitats. Such comparisons could improve sampling effectiveness and make additional data available to assess ecological quality.

## Introduction

Rivers and streams in the Mediterranean area are characterized by high seasonal and inter-annual hydrological fluctuations and often undergo surface water flow discontinuities resulting in stagnant pools or complete drying during summer (García-Roger et al., 2011; Arenas-Sánchez et al., 2021; Bonada & Resh, 2013). Moreover, a high percentage of Mediterranean streams are characterized by narrow beds and coarse substrates with boulders and bedrock (Voreadou, 1993). Ecological integrity and aquatic biodiversity in rivers are negatively impacted by climate change, increasing water demand, hydrological fluctuations, and increased pollution loads (Barceló & Sabater, 2010; Bonada & Resh, 2013; López-Doval et al., 2013; Karaouzas et al., 2018). Biomonitoring can be used to identify changes in ecological communities and the causes of those changes. In Europe, the Water Framework Directive (WFD, Directive 2000/60/EC) requests that all Member States (MS) should ensure that all surface waters are of Good Ecological Status (GES) for Natural waterbodies or Good Ecological Potential (GEP) for Heavily Modified Water Bodies and Artificial Water bodies by 2027 at the latest. Water bodies are assessed by physicochemical, hydromorphological, and biological (such as phytoplankton, macrophytes, phytobenthos, benthic macroinvertebrates, and fish) quality elements.

Benthic macroinvertebrates are a crucial component of aquatic ecosystems and have been widely used as indicators for over a century globally (Barbour et al., 1999; Bonada et al., 2006; Cummins et al., 2004; Morse et al., 2007). They are considered early- and late-warning indicators because they have short generation times, ranging from weeks to months, and can respond to environmental changes (Hering et al., 2006). Moreover, some species have life cycles of a year or more which can also be useful for documenting stressors. The use of macroinvertebrates is highly recommended in Mediterranean streams because benthic macroinvertebrates can rapidly recolonize the stream bottom in intermittent and permanent streams with strong water flow fluctuations. The macroinvertebrate community can also recover rapidly from severe or prolonged droughts and intense flooding commonly observed in the Mediterranean area (Bonada et al., 2006, 2007; Statzner & Bêche, 2010; García-Roger et al., 2013).

Biomonitoring methods can be quantitative, semi-quantitative, or qualitative, depending on the technique used. Thus, different sampling methods have been used to sample benthic macroinvertebrates based on the typology (wadeable or not-wadeable), experimental approach (qualitative or quantitative), and cost- and time-related issues (Doretto et al., 2020; Hauer & Resh, 2017). Concerning sampling tools and methods, WFD requests MS to comply with international standards (e.g., ISO 5667–3:2018, ISO 10870:2012) or other national and international standards to ensure that data are comparable and of equivalent scientific quality. In rivers, the semi-quantitative kick-sweep method, which collects samples over a defined time, is commonly used, but the total sample area is unknown (Murray-Bligh, 1999; ISO 10870:2012). In contrast, quantitative sampling provides a more precise measure of benthic macroinvertebrate abundance and diversity than semi-quantitative methods because the sample area is defined (Carter & Resh, 2001).

In Greece, the sampling method established in the WFD framework for the assessment of ecological quality is based on benthic macroinvertebrates and focuses on the sampling of all available microhabitats using a D-frame net sampler (ISO 10870:2012). This method consists of a semi-quantitative, 3-min kick and sweep method (Armitage & Hogger, 1994), with an additional minute added if submerged or if emergent riparian vegetation is present (Kemitzoglou, 2004; Wright, 2000). This sampling protocol was also used to develop the Greek index, the Hellenic Evaluation System 2, HESY2 (Lazaridou et al., 2018). The HESY2 index is the official national index for implementing the WFD in Greece. Before the adoption of WFD, another stream sampling method using a shovel (scoop like) quantitative sampler had been adopted as early as 1987 in southern Greece (island of Crete) (Voreadou, 1993). The selection of the above sampling method was not an easy procedure since several sampling apparatuses had been tested before (e.g., Surber sampler, corers, nets, Hess sampler, and grabs) in order to determine which was best suited for the peculiar river habitats of Crete. A high percentage of these streams are intermittent, with many of them characterized as having high gradient, narrow channels, turbulent flow, large boulders, and the presence of bedrock 5–10 cm below the riverbed. Through this exhaustive analysis, the shovel (scoop like) sampler, hereafter referred to as “Cretan shovel,” proved to be the most effective since (a) it is light, small, and therefore can be used by a single operator; (b) due to its small size, it can easily be pushed by hand to sample narrow spaces between large boulders, to a depth of about 7 cm, slightly disturbing the river bed to minimize the loss of motile fauna (Hynes, 1970; Macan, 1958; Peckarsky, 1984); (c) bottom sampling is possible in stony substrates with stones up to 10 cm and a water column depth up to 30 cm. A very similar sampler was first described as early as 1955 (Dittmar, 1955), then in 1977 (Prater et al., 1977) and is still in use in several sampling programs (Lorenz, 2021; Meier et al., 2006) all over the world.

Regarding these two sampling methods, we hypothesize that they could be used interchangeably to assess the river ecological quality in Greece. Thus, in the present study, we compared the benthic macroinvertebrate communities collected at six streams in Greece using two different sampling methodologies: the ISO-certified semi-quantitative kick-sweep using a D-frame net and the quantitative method using the Cretan shovel. We investigated the relationship between community metrics, functional feeding groups, and locomotion types, representing the faunal communities’ structure of the studied ecosystems between these two methods. Moreover, we compared the ecological quality of the studied streams, assessed by the HESY2, to test the assumption that biomonitoring scores are comparable. Such methodologies’ comparisons are essential since they could improve sampling effectiveness and make additional historical data available for assessment.

## Material and methods

### Sampling techniques

Samples were collected using both methods during spring on the same day and at the same site. Sites were selected for their similarity of in-stream habitat composition over the sampled reach. They were divided into shovel and D-net areas, each with comparable proportions of the major habitat types. Το reduce sampling sequence bias at the different survey sites, each sample collected by the shovel was followed a few meters upstream by the D-net sample.

**Fig. 1:**
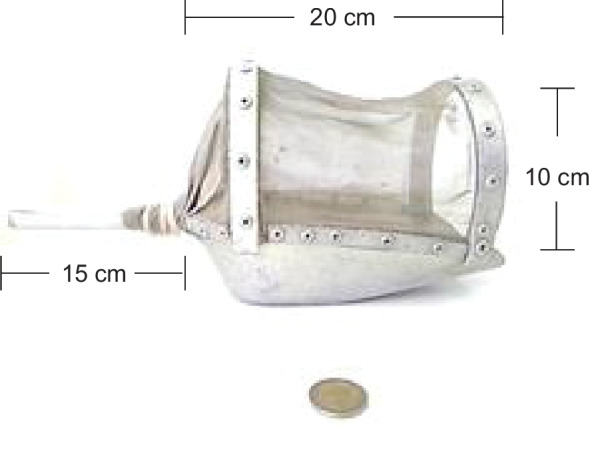
The Cretan shovel sampler. Dimensions are indicated (10 cm × 20 cm, handle: 15 cm)

Two replicate shovel samples were collected from each riffle, run and pool microhabitat (i.e., six samples from each site). The Cretan shovel sampler is 10 cm wide and 20 cm long with a sampling surface area of 400 cm^2^ and a 15 cm handle. Its opening is covered by a stainless-steel mesh with a 0.3 mm opening size which is kept stable with two strips of galvanized iron (4 mm thick) running across the front and the back top of the shovel (the front strip is 12 cm high from the bottom of the shovel, while the back one is 17 cm high) (Fig. 1). The shovel is pushed by hand into a riffle, run, and pool microhabitats to a depth of about 7 cm which slightly disturbs the sediment and dislodges benthic invertebrates, such that they carried by the river flow into the shovel. Larger stones are brushed free of animals and returned to the river bed.

Samples were collected with the D-frame net, which applied the semi-quantitative 3 min kick-sweep method (Armitage & Hogger, 1994), plus a 1 min scan of riparian vegetation (Kemitzoglou, 2004; Wright, 2000). This sampler consists of a D-shaped net (pond net) with a frame area of 575 cm^2^ (25 cm × 23 cm), a mesh opening of 0.9 mm and a depth of 27.5 cm, as well as a 150 cm handle (ISO 10870:2012). The net is placed opposite the river flow, and the operator disturbs the habitat upstream of the sampler for 3 min to dislodge macroinvertebrates that are then swept by the current into the net. During the 3 min sampling, all microhabitats are covered proportionally according to the matrix of possible river habitats (Lazaridou et al., 2018; modified from Chatzinikolaou et al., 2006).

After sampling, the processing of samples was similar regardless of the sampling method. The samples were carefully sieved with a 0.5 mm mesh size and then preserved in 80% ethanol. The identification of macroinvertebrates was to the family level except for Ostracoda, Hydracarina, Araneae, and Oligochaeta (apart from Tubificidae), as required for the application of the ecological quality index, and the abundance of each taxon was enumerated. Regarding the D-frame net, all the habitats sampled were aggregated in one sample at the site during sampling. The six replicate Cretan shovel samples were treated separately and then were aggregated into one sample before calculating community metrics, diversity indices, traits, ecological quality index, and ecological quality index metrics in order for the two sampling techniques to be comparable.

### Study area

**Fig. 2:**
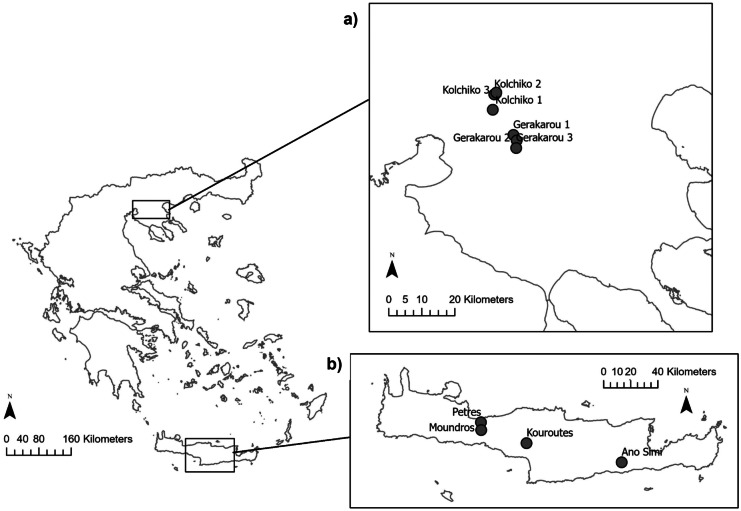
Location of sampling sites in (**a**) northern and (**b**) southern Greece

Macroinvertebrate sampling was conducted on six streams at 10 sites with less than 50 cm of depth in northern and southern Greece (Fig. 2). Specifically, we surveyed three sites from each of the two permanent streams, Kolchiko and Gerakarou, in northern Greece and one site from each of the four following streams in southern Greece (island of Crete): Petres and Ano Symi (permanent streams) and Moundros and Kouroutes (intermittent streams). These sites were chosen to provide a range of different hydromorphological characteristics (Table 1). In each location, the substrate composition was determined according to Wentworth’s (1922) grain size classification (Table 1).

**Table 1:** River type and hydromorphological characteristics of the studied sites

Site names	River type	Catchment area (km^2^)	Altitude (masl)	Sediment composition (%)						
				**Bedrock**	**Boulder**	**Cobble**	**Pebble**	**Gravel**	**Sand**	**Silt and clay**
Kolchiko_1	R-M1	79	141	0	5	20	30	15	20	10
Kolchiko_2	R-M1	82	125	0	5	10	30	15	10	30
Kolchiko_3	R-M2	115	79	5	30	30	15	5	5	10
Gerakarou_1	R-M1	20	175	0	10	20	20	10	30	10
Gerakarou_2	R-M1	28	116	0	5	20	15	20	30	10
Gerakarou_3	R-M1	48	93	0	0	25	30	20	20	5
Petres	R-M2	121	120	0	5	25	20	40	10	5
Moundros	R-M5	16	280	30	30	10	10	10	5	8
AnoSymi	R-M4	2.2	890	20	20	18	30	8	5	3
Kouroutes	R-M5	3	510	40	10	20	10	10	5	10

### Community metrics, diversity indices, and traits

Sampling techniques were compared using a set of ecological and community metrics calculated for each site. Total abundance (expressed as individuals per m^2^ only for shovel samples) and taxa richness were quantified, as well as the richness of EPT (Ephemeroptera, Plecoptera, and Trichoptera) taxa, percentage of EPT abundance, and Shannon and Pielou’s evenness indices. These metrics are routinely used in benthic ecology and are often included in biomonitoring programs (Bo et al., 2017; Burgazzi et al., 2018; Doretto et al., 2018, 2020; Sánchez-Montoya et al., 2010).

Trait metrics were also used to compare methods by assigning functional feeding groups (FFGs) and locomotion types (LTs). The FFGs were based on invertebrate morphological and behavioral adaptations for acquiring their food (Cummins & Klug, 1979; Merritt et al., 2008) and were categorized as follows: grazers and scrapers, miners, xylophagous, shredders, gatherers/collectors, active filter feeders, passive filter feeders, predators, and parasites (Moog, 1995; Schmedtje & Colling, 1996). The LTs used for the analysis were swimming/skating, swimming/diving, burrowing/boring, sprawling/walking, (semi) sessil, and others (e.g., climbing) (Schmedtje & Colling, 1996). Each taxon was assigned in a 10-point system according to its affinity to each trait category (Moog, 1995). Taxa with a score of more than five were included further in the analysis (Table A1), while the rest of the taxa were excluded to avoid taxa with low scores and high abundances. The relative abundance (%) of each FFG and LT was calculated from their total abundance for each sample. All community metrics, diversity indices, FFGs, and LTs were calculated using the ASTERICS Software (version 4.0.4).

### Ecological quality assessment

The ecological quality of the studied sites was assessed using the Hellenic Evaluation System 2 (HESY2), which is based on two metrics [i.e., Hellenic Evaluation Score (HES) and Average Hellenic Evaluation Score (AHES)] (Lazaridou et al., 2018). In the HESY2 index, taxa are categorized as sensitive, medium, and tolerant based on their tolerance to organic pollution. A specific score is assigned to each taxon, and more precisely, the score of the sensitive taxonomic groups increases with their relative abundance, while the score of the tolerant groups decreases (Lazaridou et al., 2018). The calculation of the final HESY2 score also considers stream habitat diversity characterized as “rich” or “poor” according to a specific microhabitat matrix (Chatzinikolaou et al., 2006). This matrix consists of 60 different types, taking into account the continuous changes inhabitat diversity due to geomorphological and climatic changes, and their impact on the benthic community (Artemiadou & Lazaridou, 2005).

According to the microhabitat matrix of the HESY2, described above, the river habitats of Crete were classified as “poor,” as most running water ecosystems in Crete are under strong “pressure” due to intense climatic and geomorphological conditions. There is low annual rainfall, recorded mainly during the winter months, while in summer, the lack of precipitation coincides with the maximum values of temperature and evaporation, leading to severe drought from June to September. At the same time, the quantities of water that penetrate the underground aquifer are greater than those retained on the surface since 45–50% of Crete is covered by calcareous substrates in the four most important mountain ranges of the island. The climatic and geomorphic attributes described above, combined with the tectonic structures common in Crete (faults, discontinuities, etc.), lead to the high percentage of intermittent streams and result in a “compression” of the life cycle of their fauna.

### Statistical analyses

Boxplots were constructed to compare the distribution of the selected community metrics, diversity indices, and traits between sampling methods. The traits with many zero values were excluded from the analysis, i.e., miners, xylophagous taxa, active filter feeders, parasites, swimming/skating, burrowing/boring, and others (e.g., climbing). For the remaining metrics, the statistical significance of their differences between the Cretan shovel and D-frame net samples was tested with ANOVA tests. In the case of non-normally distributed data, a non-parametric Wilcoxon rank test was performed instead. The normality of the data was assessed with the Shapiro–Wilk test, and the homogeneity of the variances was verified with Levene’s test.

Pearson correlation and linear regression analysis were used to compare normally distributed Cretan shovel and D-frame net data for community metrics, diversity indices, traits, ecological quality index, and its metrics (HES, AHES). Where data were not normally distributed, Spearman correlation was applied. If the null hypothesis is met and both sampling methods provide identical information, the *R*^2^ should equal 1 and the data should fall on the 1:1 line (i.e., *y* = *x*).

Additionally, ANCOVA tests were run to examine the relationship between community metrics, functional groups, and the substrate index (SI) (modified by Harding et al., 2009), as well as if there was an interaction between SI and the sampling techniques’ effect on the selected metrics. For the above analysis, SI was calculated using the percentages of each substrate class and summing weighted substrate percentages as follows:

$$\mathrm{SI}=\left(0.08\right)\;\%\;\mathrm{bedrock}+\left(0.07\right)\;\%\;\mathrm{boulder}\;+\left(0.06\right)\;\%\;\mathrm{cobble}\;+\left(0.05\right)\;\%\;\mathrm{pebble}\;+\left(0.04\right)\;\%\;\mathrm{granule}\;\mathrm{gravel}\;+(0.03)\;\%\;\mathrm{fine}\;\mathrm{sediment}$$

This index provides a measure of the coarseness of the substrate and varies from 3 (100% fine sediment) to 8 (100% bedrock). All the above statistical analyses were carried out with the software R (R Core Team, 2017).

Non-metric multidimensional scaling (NMDS) was implemented to evaluate differences in benthic macroinvertebrate community structures between sampling methods (PRIMER, Version 7, Clarke & Gorley, 2006). A one-way analysis of similarities (ANOSIM) of macroinvertebrate community compositions was also carried out to identify significant differences between sampling methods using 9999 permutations (PRIMER, Version 7, Clarke & Gorley, 2006). In this analysis, high *R* values (> 0.75) indicate that groups are well separated from each other, whereas low values (*R* < 0.25) indicate poor separation and little to no existing differences (Clarke & Warwick, 2001). We also performed a permutation univariate analysis of variance (PERMANOVA) (Anderson, 2001) (with α set at 0.05; 9999 permutations) to test the hypothesis of no differences between sampling methodologies. Additionally, we applied analysis of multidimensional dispersion (PERMDISP, PRIMER version 7) (Anderson et al., 2008) to compare the homogeneity of multivariate dispersion of benthic macroinvertebrates communities from the different sampling approaches. This analysis compares the average dissimilarity of samples to their group centroid (i.e., sampling methodology) based on an F statistic while calculating significance levels using permutation of least-squares residuals (9999 permutations) (Anderson et al., 2008). All the above analyses were based on a Bray–Curtis similarity matrix of presence/absence data to account for differences in sampling methodologies to conserve the whole community.

## Results

### Benthic macroinvertebrate community composition

**Fig. 3:**
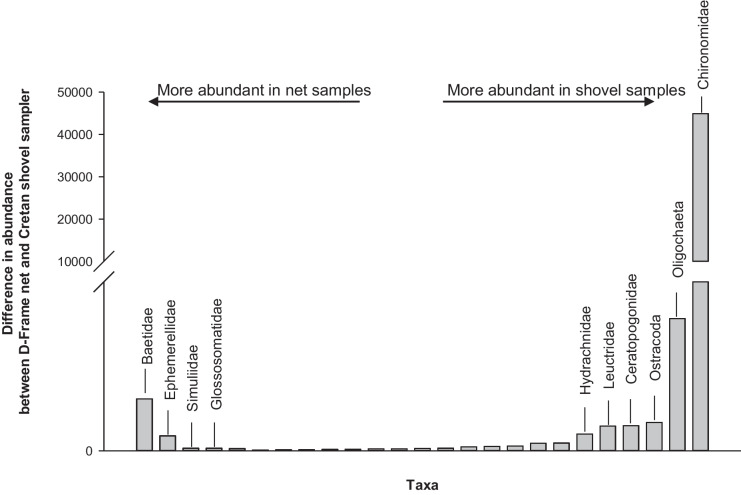
The difference in abundance between D-frame net and Cretan shovel samples for benthic macroinvertebrate taxa, aggregated across all sampling sites. Taxa with large differences between sampling methodologies are labeled. Note that only taxa (*n* = 25) with more than 16 individuals are included

In total, 62,755 of benthic macroinvertebrate individuals were collected from all sampling sites (*n* = 10) across both sampling techniques, representing 56 taxa (Table A2). The highest number of taxa (48 taxa) was recorded in D-frame net samples, followed by the Cretan shovel (44 taxa). The most abundant taxa collected using the Cretan shovel sampler were Chironomidae and Oligochaeta (Fig. 3). Additionally, the abundance of Ostracoda and Ceratopogonidae was almost five times more in shovel samples (Fig. 3). In contrast, D-frame net samples recorded a higher abundance of Baetidae and Ephemerellidae than the Cretan shovel samples (Fig. 3).

**Fig. 4:**
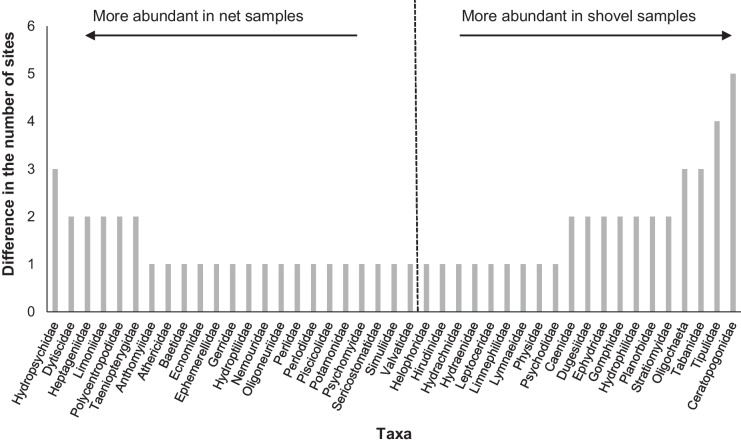
The difference in the number of sites where taxa were collected between D-frame net and Cretan shovel samples. Taxa recorded in both methodologies were not included in the graph

Concerning taxa richness, D-frame net samples recorded 11 taxa not found in Cretan shovel samples, whereas the latter collected eight taxa not captured with the net sampler (Table A2). However, for both samplers, those taxa were only recorded at one or two sites. Regarding the frequency of sampled families, the D-frame net collected more Hydropsychidae (n = 3 sites), while Cretan shovel more Ceratopogonidae and Tipulidae (5 and 4 sites, respectively) (Fig. 4).

### Community metrics, diversity indices, and traits measures

**Fig. 5:**
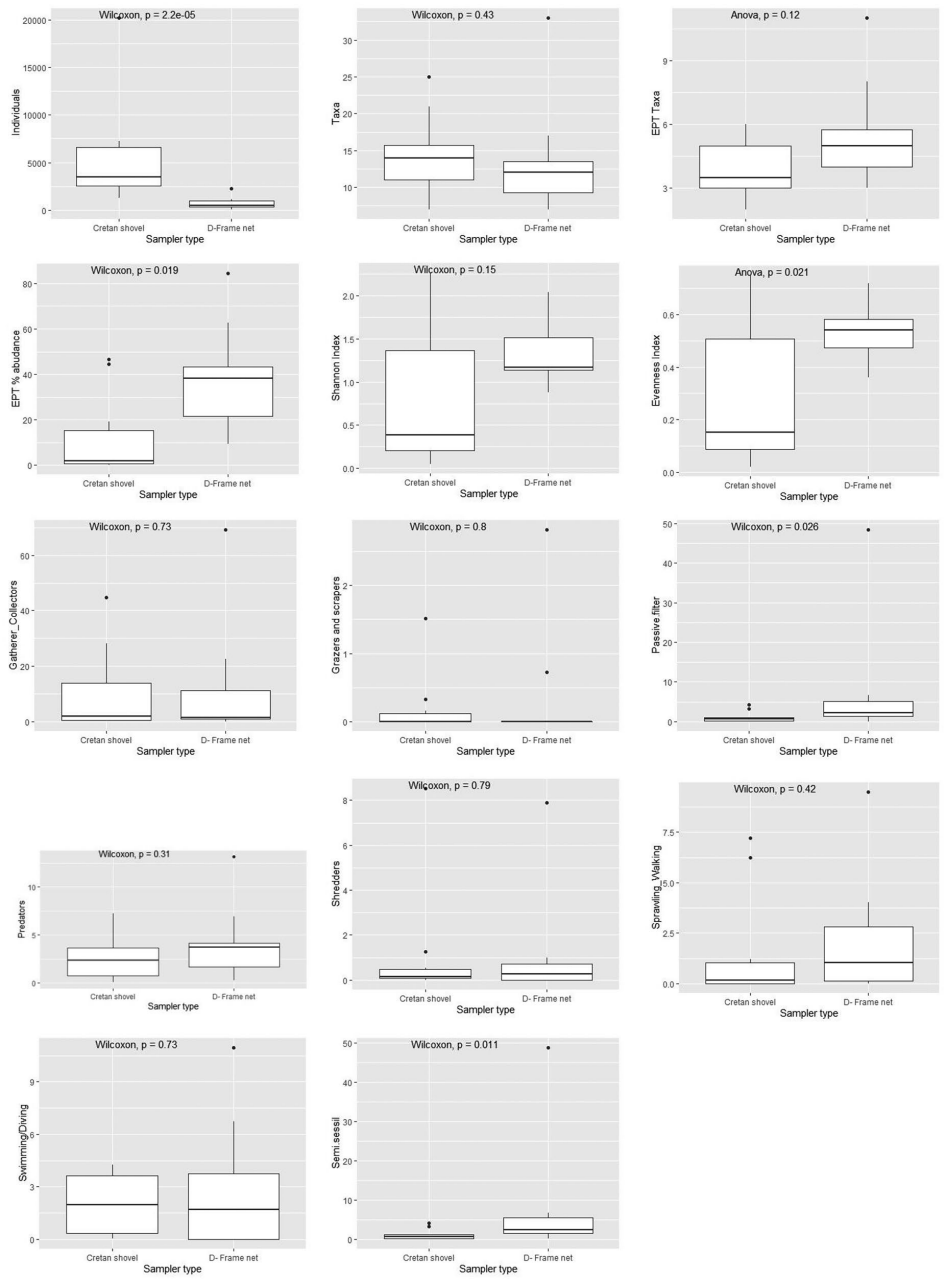
Boxplots of macroinvertebrate total abundance, taxa richness, EPT (Ephemeroptera, Plecoptera, Trichoptera) taxa, EPT relative abundance (%), Shannon, Pielou’s evenness indices, functional feeding groups, and locomotion types for each sampling method in the studied sites. Boxplots show the maximum and minimum values as well as the interquartile ranges (25–75%), with solid lines representing median values. The *p*-values indicate the statistical significance of the differences between the effectiveness of the two sampling methods

The difference between total invertebrate abundance for D-frame net and Cretan shovel samples was statistically significant (*p* < 0.05; Fig. 5), with the shovel sampler collecting more individuals. In contrast, non-significant differences were observed for the taxa and EPT richness (*p* > 0.05; Fig. 5). The EPT relative abundance (%) differed statistically between sampling techniques (*p* < 0.05) with the D-frame net sampler collecting proportionally more EPT individuals than the Cretan shovel (Fig. 5). As for Shannon diversity, there was no significant difference between the samplers (*p* > 0.05; Fig. 5), while a significant correlation was observed for the Pielou’s evenness index (*p* < 0.05; Fig. 5). Finally, concerning LGs and FFGs, there was a significant difference observed in the passive filterers group and (semi) sessile group (Wilcoxon test, *p* < 0.05; Fig. 5).

**Fig. 6:**
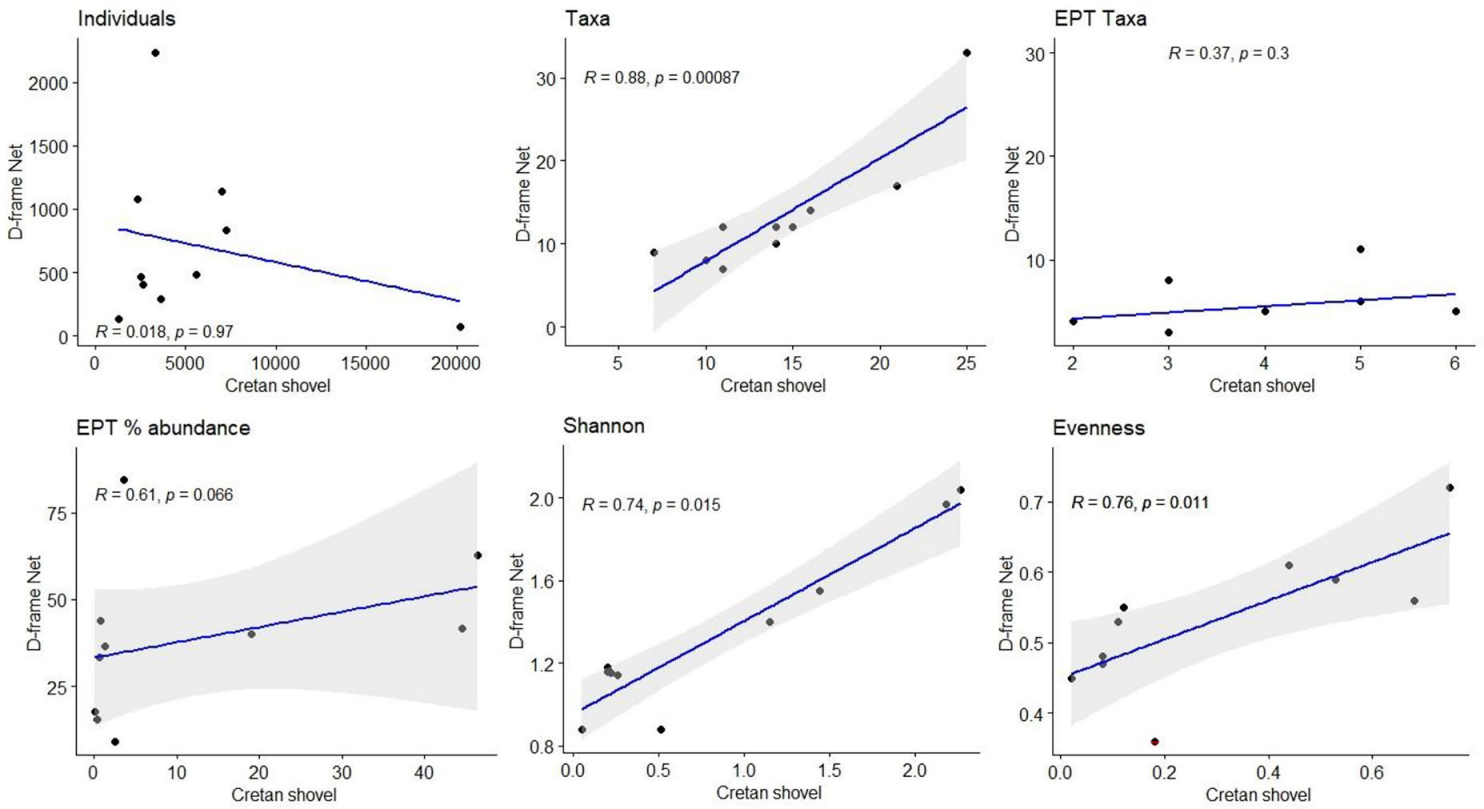
Scatter plot depicting the correlation (and 95% confidence interval of the regression line) between the total abundance, taxa richness, EPT (Ephemeroptera, Plecoptera, Trichoptera) taxa, EPT relative abundance (%), and Shannon and Pielou’s evenness indices collected in D-frame net samples versus Cretan shovel samples

The correlation between the abundance of benthic invertebrates from the samplers was low (*R*_s_ =  + 0.018, *p* > 0.05; Fig. 6), but at sites where the Cretan shovel sampler collected high numbers of taxa, the equivalent D-frame net samples were also characterized by high richness. Hence, the relationship between taxa richness for the D-frame net and Cretan shovel samples was significantly positively correlated (*R*_s_ =  + 0.88, *p* < 0.05) (Fig. 6). However, the EPT richness and EPT abundance from the samplers was not significantly correlated (*R*_s_ =  + 0.37, *p* > 0.05; *R*_s_ =  + 0.61, *p* > 0.05, respectively; Fig. 6). The two samplers for both the Shannon and Evenness indices were significantly positively correlated (*R*_s_ =  + 0.74; *p* < 0.05 and *R*_s_ =  + 0.76; *p* < 0.05, respectively; Fig. 6).

**Fig. 7:**
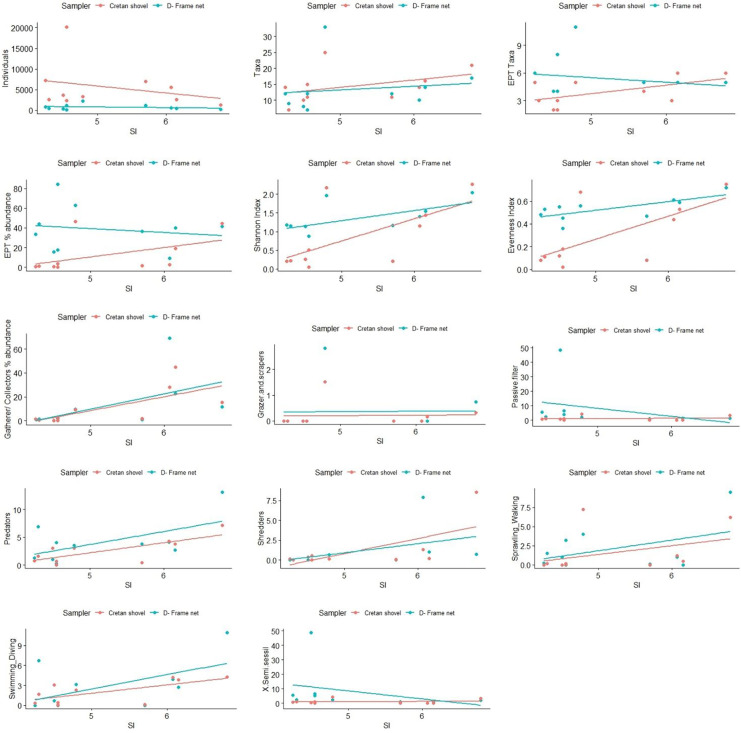
Variation of macroinvertebrate total abundance, taxa richness, EPT (Ephemeroptera, Plecoptera, Trichoptera) taxa, EPT relative abundance (%), Shannon, Pielou’s evenness indices, functional feeding groups, and locomotion types between D-frame net samples versus Cretan shovel samples in relationship to substrate index (SI)

The ANCOVA test did not show an interaction between the SI index and sampler type for any metric or diversity index (Fig. 7). Shannon index increased significantly as the SI index increased (ANCOVA, *F* = 9.58, *df* = 1.16, *p* < 0.05); this pattern was similar for the two samplers (*F* = 3.9, *df* = 1.16, *p* = 0.06). However, except for high SI index values, the Shannon values for D-frame net samples were more diverse than Cretan shovel samples. In contrast, the evenness values increased significantly as SI index increased (ANCOVA, *F* = 11.82, *df* = 1.16, *p* < 0.05), and the pattern was different between the two samplers (ANCOVA, *F* = 10.72, *df* = 1.16, *p* < 0.05). The shovel samples were characterized by low evenness values at low SI values. Among the FFGs and LTs used, the SI index was significant for the gatherers-collectors and shredders (ANCOVA, *F* = 8.08, *df* = 1.16, *p* < 0.05 and *F* = 14.61, *df* = 1.16, *p* < 0.05, respectively), while the sampler type had a significant effect in passive filterers and swimming/diving group (ANCOVA, *F* = 4.6087, *df* = 1.16, *p* < 0.05; *F* = 6.0438, *df* = 1.16, *p* < 0.05).

**Fig. 8:**
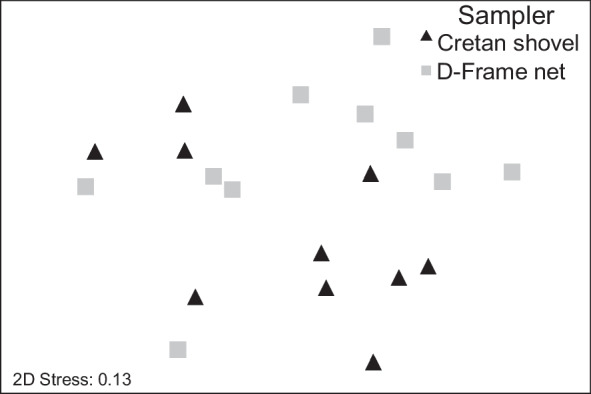
NMDS plot of benthic macroinvertebrates from D-frame net and Cretan shovel samples

The NMDS analysis combined with ANOSIM (*R* = 0.02) indicated no differences between sampling methodologies in benthic macroinvertebrate communities (Fig. 8). Additionally, PERMANOVA showed no significant difference in benthic macroinvertebrate composition between sampling methodologies (*F* = 1.083, *p* > 0.05). The PERMDISP also identified no significant differences in the homogeneity of spatial dispersion in macroinvertebrate community structure between the two samplers (*F* = 0.124, *p* > 0.05).

### Assessment of ecological quality

Differences between the HESY2 ecological quality assessments were observed among the D-frame net and Cretan shovel samples in four out of ten studied sites (40%) (Table 2). In the Cretan streams, only one site (Ano Symi) had a different ecological category between the two methods (Table 2), while three sites were different in northern Greece (Gerakarou sites, Table 2). Specifically, the Cretan shovel samples were assigned to a lower class category in three northern sites, whereas the reverse was observed in one site in Crete (Table 2). As the scoring index is continuous, classification could be altered by small increments in the score if they fall close to the class boundary. In our case, the ecological quality scores of these sites were close to boundaries [i.e., −0.06 for Gerakarou sites (class interval: 0.25) and −0.05 for Ano Symi (class interval: 0.21)] (Table 2). In Gerakarou sites, the differences in ecological quality are attributed to the absence or the low contribution of sensitive taxa collected by the Cretan shovel. On the contrary, in Ano Symi, the Cretan shovel collected two more sensitive taxa (i.e., Philopotamidae, Taeniopterygidae, Table A2), and the relative abundance of two sensitive taxa was higher (i.e., Cretan shovel: Limnephilidae 1.3% and Nemouridae 8.5%; D-frame: Limnephilidae 0.7% and Nemouridae: 0.7%). Thus, in these sites, the observed scores were higher following the increase in the relative abundance of sensitive taxa.

**Table 2:** Results from sensitive, medium, and tolerant to pollution taxa (%), HESY2, and its metrics estimated using D-frame net and Cretan shovel samples

Sample	Sampling method	% Sensitive taxa	% Medium taxa	% Tolerant taxa	HES	AHES	HESY2	Quality HESY2
**Petres**	Cretan shovel	17.4	8.9	73.7	1316	52.64	1.00	High
	D-frame net	16.4	8.5	75.1	1720	52.12	1.00	High
**Moundros**	Cretan shovel	2.5	1.5	95.5	879	58.60	0.90	Good
	D-frame net	4.9	2.2	92.2	667	51.31	0.70	Good
**AnoSymi**	Cretan shovel	43.3	8.2	48.5	1,212	57.71	1.00	High
	D-frame net	47.4	11.7	40.9	921	54.18	0.80	Good
**Kouroutes**	Cretan shovel	2.5	4.1	93.4	642	45.86	0.70	Good
	D-frame net	9.1	3.7	87.2	494	49.40	0.70	Good
**Kolchiko_1**	Cretan shovel	0.3	9.7	99.0	530	48.18	0.56	Moderate
	D-frame net	3.5	1.7	94.8	570	47.50	0.56	Moderate
**Kolchiko_2**	Cretan shovel	0.4	1.1	98.5	660	47.14	0.56	Moderate
	D-frame net	1.3	5.9	92.8	636	53.00	0.56	Moderate
**Kolchiko_3**	Cretan shovel	0.0	0.1	99.9	410	37.27	0.44	Poor
	D-frame net	4.1	5.4	90.5	308	44.00	0.33	Poor
**Gerakarou_1**	Cretan shovel	0.0	2.0	97.1	578	41.29	0.44	Poor
	D-frame net	4.3	6.8	88.8	690	57.50	0.67	Moderate
**Gerakarou_2**	Cretan shovel	0.2	2.8	97.0	334	47.71	0.44	Poor
	D-frame net	1.7	8.7	89.6	466	51.78	0.56	Moderate
**Gerakarou_3**	Cretan shovel	0.0	3.8	96.1	354	39.33	0.44	Poor
	D-frame net	2.1	48.8	49.1	437	54.63	0.67	Moderate

*HES* Hellenic Evaluation Score; *AHES* Average Hellenic Evaluation Score; *HESY2* Hellenic Evaluation System

**Fig. 9:**
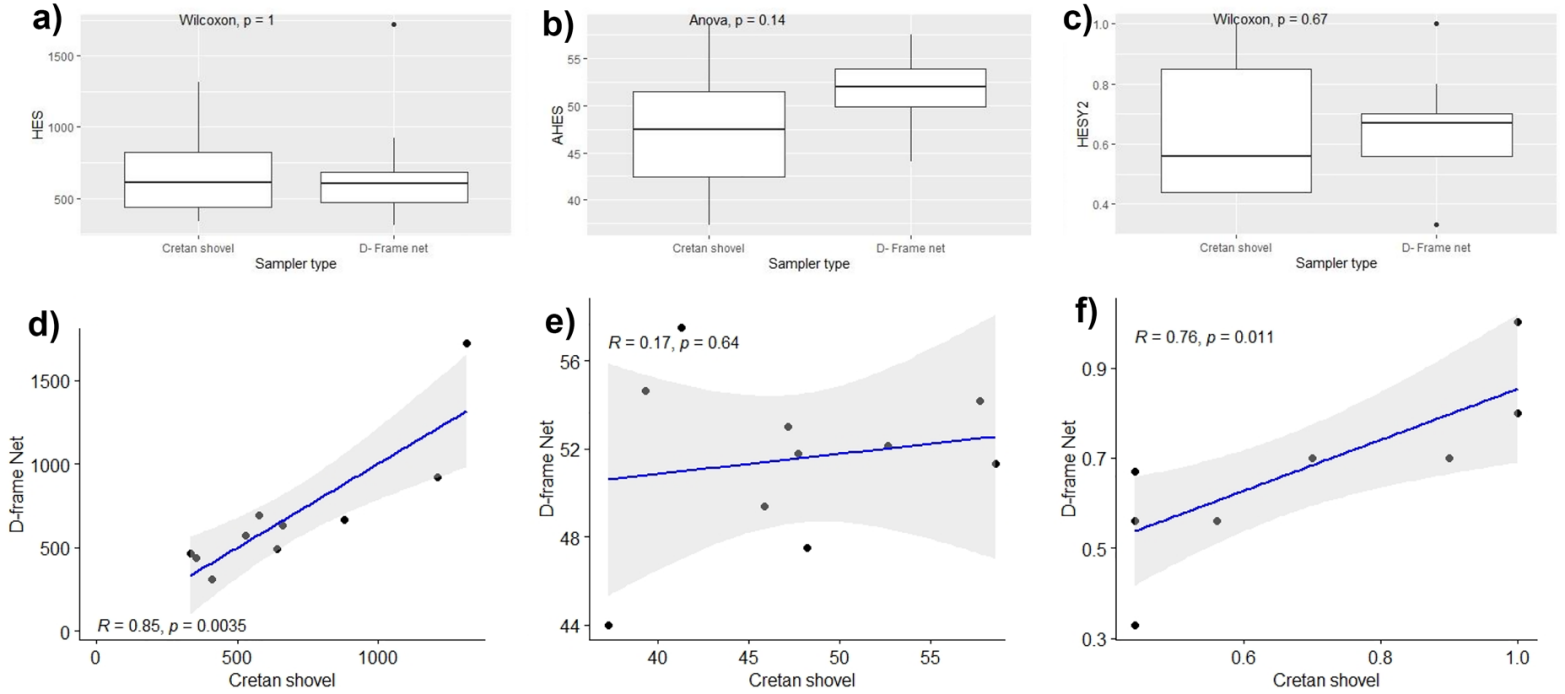
(**a**–**c**) Boxplots of HES (hellenic evaluation score), AHES (average hellenic evaluation score), and HESY2 (hellenic evaluation system 2) for each sampling method in the studied sites. Boxplots show the maximum and minimum values as well as the interquartile ranges (25–75%), with solid lines representing median values. (**d**, **e**) Scatter plots depicting the correlation (and 95% confidence interval of the regression line) between the HES, AHES, and HESY 2 calculated from D-frame net samples versus Cretan shovel samples. The *p*-values indicate the statistical significance of the differences between the effectiveness of the two sampling methods

However, no statistical differences between the two samplers were found when box plots of HES, AHES, and HESY2 were examined (Fig. 9a–c). In contrast, a correlation was identified between the two samplers for HES and HESY2, although there was no significant correlation between the samplers for AHES (*R* = 0.17, *p* > 0.05) (Fig. 9d–f).

## Discussion

This study aimed to compare two benthic macroinvertebrate sampling methodologies in Greek rivers: the D-frame net and the Cretan shovel sampler. We attempted to determine differences between the D-frame net and Cretan shovel sampler and the advantages of each for describing benthic macroinvertebrate communities for assessment purposes. Our findings indicate that the two different sampling methods collect different abundances of benthic macroinvertebrates, although the composition of communities did not differ significantly, as demonstrated by the NMDS plot. Concerning community metrics, it seems that both samplers collected approximately the same number of taxa (i.e., D-frame net: 48 taxa and Cretan shovel: 44 taxa), EPT taxa (i.e., D-frame net: 19 taxa and Cretan shovel: 14 taxa), while the shovel sampler collected more individuals. Other similar quantitative samplers, like the Surber sampler, also collect more macroinvertebrate individuals (Everall et al., 2017; Ghani et al., 2016), which may be due to the specific surface area and the greater intensity of Surber and shovel sampling (Macan, 1958; Storey, 1991). A disadvantage of greater macroinvertebrate abundance is that it requires more time and effort for sorting and identification.

The highest Shannon diversity index values were recorded with the D-frame net, but these values did not differ significantly between the two samplers. In contrast, Pielou’s evenness index showed a significant difference between the D-frame net and shovel sampler. In shovel samples, this index had a wide range of values (0.02–0.75) with the lower ones recorded in the northern streams. This difference could be related to the higher percentage of fine sediment composition (sand, silt, and clay) in the studied streams of northern Greece compared to southern Greece.

The EPT abundance was statistically different between the two methods, while EPT taxa richness was not. Although not significant, the Cretan shovel collected higher numbers of EPT taxa than the D-frame net. This was especially true of Baetidae, the most abundant family among the EPT taxa in our study. Baetidae is a good representative of motile organisms, as they are quite good swimmers compared to other Ephemeroptera, which are clingers or borrowers (Bouchard, 2004). It is already known that net samplers collect motile benthic macroinvertebrates more effectively, while shovels are less effective (Hynes, 1970; Macan, 1958). Shovels are forced into the substrate to the desired depth and then pushed a distance forward, shifting thus the substrate. As the shovel blade passes under it, some motile organisms can swim upstream and out of the sampler (Macan, 1958). Another reason could be the width of the front opening of the shovels, which usually ranges from 22.5 cm (Macan, 1958) to 23 cm (Dittmar, 1955) and 25 cm (Lorenz, 2021). The Cretan shovel’s front opening is only 10 cm, facilitating motile groups to escape.

The opening of the Cretan shovel could be broader as a larger shovel will reduce the sampling error (Prater et al., 1977; Schwoerbel, 1970). However, the most suitable area to be sampled with a shovel was found to be 400 cm^2^, as a larger sampler was too unwieldy, especially in a strong current (Percival & Whitehead, 1929). Moreover, a larger sampler would need two operators (Schwoerbel, 1970). It is preferable that a single operator can handle the sampler because a second operator may disturb the stream bed above the sampler to dislodge animals (Macan, 1958). Additionally, in streams characterized by narrow beds and high percentages of coarse substrates, as in Crete, a larger shovel sampler would be difficult to deploy due to the small spaces between large boulders.

Regarding macroinvertebrate traits, passive filter feeders from the FFGs and (semi) sessile organisms from LTs were found more in D-frame net samples. The passive filter feeders included Oligoneuridae, Philopotamidae, and Simuliidae, and the (semi) sessils included Ecnomidae, Hydropsychidae, Philopotamidae, Polycentropodidae, and Simuliidae. The family Simuliidae was the most abundant, and most were captured in the D-frame net. Simuliidae are restricted to a narrow habitat type (De Moor et al., 1986) which is coarse, exposed substrates. This habitat is more effectively sampled by the D-net method, while the shovel sampler is less effective because it usually samples small stones (up to 342 7–8 cm), gravel, sand, and mud (Percival & Whitehead, 1929; Macan, 1958; Schwoerbel, 1970; Elliot et al., 1980).

Concerning the HESY2 index, four out of ten sites (40%) had a different ecological quality. In northern Greece, the Cretan shovel samples were assigned in a lower class category than the D-frame net sampling at three sites (30%) in the same stream, whereas the reverse was observed only in one stream in the south. In the three northern sites, the D-frame net, compared to the Cretan shovel, collected more sensitive taxa according to the categorization of HESY2. This could be explained by the fact that the Cretan shovel sampler collects fewer motile organisms, and as a result, the category assigned to each sample may be of a lower quality according to the HESY2. The latter is crucial if the sites are assigned to the moderate category rather than the good one, as the WFD will require more measures and money to improve their quality category. The class category assigned to the Cretan shovel samples was comparable to the D-frame net ones in 60% of cases. It seems that the Cretan shovel is efficient in streams with less than 30 cm depth and coarse, narrow, and turbulent river bed (characterizing the island streams). Instead, the D-frame net appears to be effective in more variable geomorphology, although, in our study, it was difficult to manipulate it in river beds with boulders.

Most of the northern sites studied had different geomorphology than the southern ones. In the north, sampling sites were located in relatively lower altitudes, resulting in wider riverbeds with a higher percentage of fine sediments. In these sites, a greater effort using the shovel sampler is probably needed to assess the ecological quality since this method requires more man-hours of sorting and identification. In the south, three of the four sampling sites were mountainous high-altitude sites with narrow riverbeds and a higher percentage of bedrock and boulders. In the riverbeds of Crete, the shovel sampling methodology (six replicates in each site) was efficient at capturing the present taxa. Only one site in Crete was located at a low altitude; there, the shovel captured less sensitive taxa than the net. Thus, if these two sampling methodologies are to be applied interchangeably to assess the ecological quality in the streams of northern Greece, more sampling effort is needed from the Cretan shovel. However, the shovel seems to be efficient in southern Cretan river habitats.

Comparing two sampling methodologies in different river habitat types found in northern and southern Greece also raises some issues regarding macroinvertebrate sampling. In Cretan streams, the assessment of ecological quality is complicated by the difficulty of efficiently sampling benthic macroinvertebrates. This occurs due to specific habitat characteristics of Cretan streams including seasonal flow, high gradients, narrow riverbeds, turbulent flow, and coarse substrates, with a high percentage of bedrock and boulders. Such streams exist not only in Crete but also on the Greek mainland as well as in the Ionian Sea, the Aegean archipelagos, and in many other locations in the Mediterranean area. The Cretan shovel methodology could be used to address the difficulty of sampling the stream habitats found in these regions. Thus, sampling comparisons and the possible effectiveness of sampling methodologies, such as the Cretan shovel, in dynamic and extreme river habitats are crucial since they could improve sampling efficiency. Finally, additional historical data from the Cretan shovel from approximately 250 sites, as early as 1987, are available to assess the ecological quality of Cretan streams and provide the opportunity to record long-term trends in these freshwater ecosystems.

## Conclusions

This study highlighted the performance of two different sampling methodologies to collect benthic macroinvertebrates in wadeable streams in Greece. Our analysis was based on a data set covering 10 sites in northern and southern Greece and included 56 taxa and 62,755 identified individuals. Most of the community metrics, diversity indices, and traits were comparable between hand net and shovel sampler. Most differences (30%) in ecological quality class were observed in the sites of northern Greece, which may be due to differences in habitat between the river habitats in the streams of northern and southern Greece. Our study indicated that the Cretan shovel is efficient for the sampling of coarse, narrow, turbulent and hydrologically fluctuating river habitats like those of Crete. Further field surveys should be conducted in other streams in northern and southern Greece, i.e., the Aegean islands. Such studies could enhance our knowledge to improve sampling procedures and develop more effective sampling strategies. Finally, the efficiency of the shovel sampler in Crete sets the opportunity to use historical shovel samples since 1987 and investigates long-term trends in Crete.

## Data Availability

The data that support the findings of this study are available on request from the corresponding author.

## Appendix

**Table A1:** Scores assigned in each taxon for each trait category (functional feeding groups and locomotion types)

Functional feeding groups									
**Taxa**	**Grazer and scrapers**	**Miners**	**Xylophagous**	**Shredders**	**Gatherer/collectors**	**Active filter**	**Passive filter**	**Predator**	**Parasites**
Anthomyiidae								10	
Athericidae								10	
Caenidae					10				
Ceratopogonidae								10	
Dixidae					3	7			
Dugesiidae								10	
Dytiscidae								10	
Empididae								10	
Gerridae								10	
Glossosomatidae	10								
Gomphidae								10	
Hirudinidae									10
Hydraenidae	10								
Limoniidae					10				
Mesoveliidae								10	
Nemouridae				7	3				
Neritidae	10								
Oligochaeta					10				
Oligoneuriidae							10		
Ostracoda				2	8				
Perlidae	1							9	
Perlodidae	1							9	
Philopotamidae							10		
Piscicolidae									10
Polycentropodidae							1	9	
Psychomyiidae				10					
Sericostomatidae				10					
Simuliidae							10		
Sphaeriidae						10			
Tabanidae								10	
Tipulidae				7	3				

Locomotion types					
**Taxa**	Swimming/skating	Swimming/diving	Sprawling/walking	Burrowing/boring	(Semi) sessil
Ceratopogonidae		7			
Dixidae			10		
Dytiscidae		6	1		
Ecnomidae					10
Gerridae	10				
Glossosomatidae			10		
Gomphidae				10	
Heptageniidae		2	8		
Hydrophilidae		2	6		
Hydropsychidae					10
Hydroptilidae			10		
Limnephilidae			10		
Lymnaeidae			10		
Mesoveliidae	10				
Philopotamidae					10
Polycentropodidae					10
Psychomyiidae			10		
Scirtidae			10		
Sericostomatidae			10		
Simuliidae	1		3		6

**Table A2:** Taxa of benthic macroinvertebrates collected at sampling sites

**Taxa**	**Cretan shovel**	**D-frame net**	**Taxa**	**Cretan shovel**	**D-frame net**
Anthomyiidae		√	Limnephilidae	√	√
Asellidae	√	√	Limoniidae	√	√
Athericidae		√	Lymnaeidae	√	
Baetidae	√	√	Mesoveliidae	√	√
Caenidae	√	√	Nemouridae	√	√
Ceratopogonidae	√	√	Neritidae	√	√
Chironomidae	√	√	Oligochaeta	√	√
Dixidae	√	√	Oligoneuriidae		√
Dugesiidae	√		Ostracoda	√	√
Dytiscidae	√	√	Perlidae		√
Ecnomidae		√	Perlodidae	√	√
Empididae	√	√	Philopotamidae	√	√
Ephemerellidae	√	√	Physidae	√	
Ephydridae	√	√	Piscicolidae		√
Gerridae		√	Planorbidae	√	√
Glossosomatidae	√	√	Polycentropodidae		√
Gomphidae	√		Potamidae		√
Helophoridae	√		Psychodidae	√	√
Heptageniidae	√	√	Psychomyiidae		√
Hirudinidae	√		Scirtidae	√	√
Hydrachnidae	√	√	Sericostomatidae		√
Hydraenidae	√	√	Simuliidae	√	√
Hydrobiidae	√	√	Sphaeriidae	√	√
Hydrophilidae	√	√	Stratiomyiidae	√	
Hydropsychidae	√	√	Tabanidae	√	√
Hydroptilidae	√	√	Taeniopterygidae	√	√
Leptoceridae	√		Tipulidae	√	√
Leuctridae	√	√	Valvatidae		√
